# Cost-Effectiveness Analysis of Trastuzumab-Emtansine as Adjuvant Therapy for HER2-Positive Early Breast Cancer with Residual Invasive Disease in Colombia

**DOI:** 10.36469/001c.156056

**Published:** 2026-02-17

**Authors:** Daniel Samacá-Samacá, Diego Ballén, Milton Lombana, Melissa Díaz-Puentes, Sergio Augusto Cáceres-Maldonado, Juliana Saavedra, Laura Prieto-Pinto

**Affiliations:** 1 Evidence Generation Roche Colombia, Bogotá, Colombia; 2 Instituto Nacional de Cancerología, Bogotá, Colombia; 3 Clinica de Occidente, Cali, Colombia; 4 Medical Affairs Roche Colombia, Bogotá, Colombia

**Keywords:** breast cancer, cost-effectiveness, HER2 positive, residual disease

## Abstract

**Background:**

Patients with HER2-positive (HER2+) breast cancer (BC) who have residual invasive disease after neoadjuvant therapy remain at a significantly increased risk of recurrence. Updated results from the KATHERINE trial demonstrated the clinical benefit of trastuzumab-emtansine (T-DM1), showing a significant improvement in event-free survival. However, in budget-constrained settings, assessing the cost-effectiveness of TDM-1 is essential to guide sustainable adoption.

**Objective:**

To evaluate the cost-effectiveness analysis of adjuvant T-DM1 compared with trastuzumab in HER2+ early BC patients and residual disease, following neoadjuvant treatment, from the Colombian Health System perspective.

**Methods:**

We conducted a Markov model with a lifetime horizon to evaluate the cost-effectiveness of T-DM1 using the updated results of the KATHERINE trial (8.4-year follow-up). The model comprises 6 health states: patients with residual invasive disease on adjuvant treatment, non-metastatic recurrence, remission after non-metastatic recurrence, metastatic disease (first-line [1LmBC] and subsequent lines [2LmBC]), and death. Direct medical costs were included. The primary outcome was quality-adjusted life-years, with both costs and effects discounted annually at a 5% rate. Model assumptions were based on current guidelines and validated by local experts. Sensitivity and scenario analysis were performed to confirm the reliability of the results. Prices are shown in 2024 US dollars (4071.35 Colombian pesos = US $1)

**Results:**

Under a willingness-to-pay threshold of 86% of the 2024 gross domestic product per capita (US 6831),T−DM1wasdominantovertrastuzumab.Costsavingsweremainlydrivenbyareductioninrecurrences(>50−30 510) and 2LmBC (US $−6317). The probabilistic sensitivity analysis confirmed T-DM1 dominance in 81.2% of the 1000 Monte Carlo simulations, demonstrating consistency across settings.

**Conclusion:**

T-DM1 is a cost-effective and dominant strategy for the adjuvant treatment of residual invasive HER2+ early BC in Colombia, driven by cost savings from reduced high-cost recurrences. These findings support the adoption of T-DM1 to improve outcomes while ensuring efficient use of healthcare resources.

## BACKGROUND

Breast cancer (BC) is the most frequently diagnosed cancer in women worldwide and remains the leading cause of cancer-related deaths. In 2022, BC accounted for an estimated 2 million new cases and more than 600 000 deaths worldwide.^1^ In Colombia, 97 740 prevalent cases were reported between 2022 and 2023, including 9484 new cases (9.7%).^2^ With a mortality rate of 13.3 per 100 000 inhabitants, BC remains the leading cause of cancer mortality among women in the country, highlighting the need for strategies that improve treatment outcomes.^1^

Among BC phenotypes, positive human epidermal growth factor receptor 2 (HER2+) accounts for 17.5% of cases and is associated with more aggressive disease and worse clinical outcomes.^2,3^ Despite advances in anti-HER2 therapies, including dual blockade with pertuzumab and trastuzumab in neoadjuvant and adjuvant settings, 40% to 60% of patients with HER2+ BC continue to have residual invasive disease after initial treatment.^3–7^

The high risk of recurrence in patients with HER2+ early BC (eBC) with residual disease prompted the development of trastuzumab-emtansine (T-DM1), an antibody-drug conjugate targeting HER2, that combines trastuzumab with the microtubule inhibitor DM1 to improve survival.^8^ T-DM1 has shown significant efficacy in reducing disease recurrence. The updated results of the KATHERINE trial (NCT01772472) showed a 46% decrease in the risk of recurrence of invasive disease or death with T-DM1 compared with trastuzumab in patients with residual invasive disease after neoadjuvant therapy.^9^

Preventing recurrences and improving long-term outcomes in BC are key for reducing the economic burden of advanced disease, particularly in the highly aggressive HER2+ phenotype.^10,11^ This is especially relevant in low- and middle-income countries, where health systems often face significant budget constraints.^12,13^ Local evidence shows that advanced HER2+ BC results in a substantially higher economic burden than early-stage disease with 5-year costs reported to be 2 to 5 times higher, underscoring the financial impact of disease progression on healthcare systems.^14^

In this context, the potential of T-DM1 to prevent recurrences may offset the escalating costs associated with advanced disease, thereby alleviating the overall financial burden on the healthcare system. While T-DM1 provides well-documented clinical benefits for patients with HER2+ eBC and residual invasive disease, its adoption may be associated with higher upfront costs. Consequently, evaluating its long-term economic value through cost-effectiveness analyses is therefore essential, as these assessments inform evidence-based decision-making and support the efficient allocation of limited healthcare resources.^15,16^ The objective of this study was to evaluate the cost-effectiveness of adjuvant T-DM1 compared with trastuzumab (Herceptin and biosimilars), in women with HER2+ eBC and residual invasive disease following neoadjuvant therapy, adopting the perspective of the Colombian General Social Security Health System (Sistema General de Seguridad Social en Salud [SGSSS]).

## METHODS

### Study Design and Model Overview

This study updates a previously published cost-effectiveness analysis of trastuzumab-emtansine (T-DM1) in Colombia^17^ by incorporating 2024 cost data, updated efficacy results from the KATHERINE trial with a median follow-up of 8.4 years,^9^ the introduction of new therapies in the metastatic setting,^18^ and an updated country-specific willingness-to-pay (WTP) threshold.^19^

A state-transition Markov model with a 1-month cycle length and half-cycle corrections was used to project outcomes over a 48-year time horizon, corresponding to lifetime, assuming a baseline age of 52 years.^20^ The analysis adopted a health system perspective, considering only direct medical costs. Costs and health outcomes were discounted at an annual rate of 5%, consistent with the guidelines of the national health technology assessment (HTA) agency (Instituto de Evaluación Tecnológica en Salud [IETS]).^21^ The WTP threshold was set at US $6831, corresponding to 86% of Colombia’s 2024 gross domestic product per capita, using an exchange rate of US $1 = 4071.35 Colombian pesos (COP), as estimated for Colombia by Espinosa et al.^19^

The cost-effectiveness analysis compared T-DM1 with trastuzumab (including IV and subcutaneous Herceptin, as well as available biosimilars) as adjuvant therapy following neoadjuvant taxane-based chemotherapy plus trastuzumab. The cost of trastuzumab was estimated using a weighted average price across all available presentations (reference biologic Herceptin and biosimilars), according to their respective market shares reported in the Colombian SISMED database. Dosing regimens reflected clinical trial protocols consisting of 14 cycles of T-DM1 at 3.6 mg/kg every 21 days, and 14 cycles of trastuzumab at 6 mg/kg per cycle, with an 8 mg/kg IV loading dose. The primary outcome was the incremental cost per quality-adjusted life-year (QALY) gained.

### Model Structure and Health States

**Figure 1.**
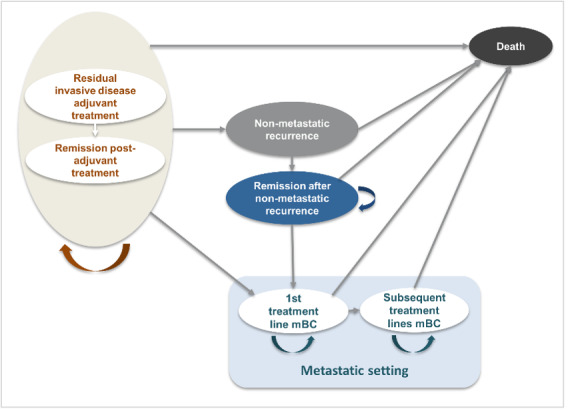
Model Structure Abbreviation: mBC, metastatic breast cancer.

The model, previously reviewed by several HTA agencies^6,22–25^ and publicly documented (eg, NICE website),^26^ was adapted to the Colombian healthcare context (**Figure 1**). The model aligns with clinical practice and reflects the disease progression of HER2+ eBC following surgery and neoadjuvant therapy. The model was executed in Microsoft Excel.

Patients entered the model in the *residual invasive disease on adjuvant treatment* state, categorized as either on treatment (adjuvant T-DM1 or trastuzumab) or off treatment (post-adjuvant therapy, alive, and disease-free, under monitoring). From this point, patients could transition to the following health states: *non-metastatic recurrence* (includes locoregional recurrence and contralateral BC), *metastatic disease* (first- and subsequent-line) or *death* (absorbing state). Progression could occur sequentially from non-metastatic recurrence to remission, or directly to death. Transition probabilities between states were informed by clinical trial data and supported by expert assumptions.

No direct transition from non-metastatic recurrence to first-line metastatic disease (1L mBC) was modeled. According to the KATHERINE trial, patients who developed a metastatic recurrence within 2 months of a locoregional or contralateral recurrence were classified as having metastatic recurrence as their initial event. Therefore, no data are available to estimate the conditional risk of metastatic progression following a non-metastatic relapse. As a result, this transition was not explicitly modeled to avoid introducing unsupported assumption, while ensuring consistency with the clinical trial definitions and estimations.

### Effectiveness Inputs

Transition probabilities for invasive disease-free survival, non-metastatic recurrence, and progression to metastatic states were derived from the KATHERINE trial (median follow-up, 8.4 years).^9^ The full treatment effect was assumed to persist until 84 months (KATHERINE trial), gradually declining thereafter, with no effect beyond 120 months,^27^ as observed in previous trials of long-term sustained HER2 suppression, where the treatment effect tends to wane near the 10-year mark. The use of the KATHERINE trial as the primary source of clinical inputs reflects its direct relevance to patients with residual invasive HER2+ eBC in the adjuvant setting; where trial data were not directly available, assumptions were informed by previously published economic evaluations and HTA-reviewed models in HER2+ eBC.^6,25,26,28^

A mixture-cure model was applied to extrapolate survival data across the lifetime horizon. Kaplan-Meier curves were digitized and fitted to standard parametric distributions (exponential, Weibull, log-normal, and log-logistic). Goodness-of-fit was assessed using Akaike and Bayesian information criteria, with the log-normal distribution ranking first in both criteria and being selected for final extrapolation. Additionally, visual inspection was performed to ensure close agreement between model-predicted survival and the observed Kaplan-Meier curves during the period of trial follow-up, providing internal validation of model fit prior to extrapolation. **Supplementary Table S1** contains additional data regarding distributions and the information criteria.

Treatment duration was informed by the observed duration of therapy for each regimen, as reported in the respective Phase 3/4 clinical trials. Probabilities for progression to and within metastatic disease were sourced from the EMILIA,^29^ DESTINY-Breast03,^18^ and CLEOPATRA trials.^30^ As no evidence was available on the sequential use of pertuzumab + trastuzumab + taxane and T-DM1 following first-line metastatic treatment, these second-line metastatic options were considered only in terms of their cost impact to minimize uncertainty. Transition probabilities, including additional health states, are presented in **Table 1**.

**Table 1.** Model Inputs

**Parameter**	**Input**	**Source**
Time horizon	48 years	Equivalent to 100 years (median age at baseline set at 52 years)
Annual discount rate (cost and effects)	5%	IETS^21^
Mean age of the cohort	52 years	Rueda et al^20^
Body weight	67 kg	Rueda et al^20^
Disease-free survival		
T-DM1	85.58%	KATHERINE trial^9^
Trastuzumab	71.11%	
Non-metastatic recurrence to remission	Automatic after 12 mo if alive	Takeuchi et al^31^
Monthly disease progression of remission after non-metastatic recurrence to 1L mBC	0.0076	Hamilton et al^32^
Monthly probability of disease progression from 1L mBC to 2L+ mBC		
T-DM1	0.0428	EMILIA^29^
T-DXd	0.0186	DESTINY-Breast03^18^
HT	0.0469	EMILIA^29^
Monthly probability of disease progression in subsequent lines mBC		
T-DXd	0.0186	DESTINY-Breast03^18^
HT	0.0315	CLEOPATRA^30^
Utility data		
Residual invasive disease (on-treatment)	0.77	KATHERINE trial^9^
Residual invasive disease (off-treatment)	0.78	KATHERINE trial^9^
Non-metastatic recurrence	0.77	KATHERINE trial^9^
Remission after non-metastatic recurrence	0.78	KATHERINE trial^9^
Metastatic health state (1L mBC)	0.77	Lloyd et al^33^
Progressed metastatic health state (2L+ mBC)	0.52	Lloyd et al^33^

Abbreviation: 1L, first line; 2L+, subsequent line; mBC, metastatic breast cancer.

### Cost Inputs

Costs included direct medical expenses for drug acquisition and administration, monitoring, supportive care, and the management of adverse events. A micro-costing approach was applied to estimate healthcare resource use, including procedures, laboratory tests, imaging, and medications. Frequencies and unit costs for healthcare resources were obtained from public sources: clinical practice guidelines and management protocols were used to identify resources, while national databases, including SISMED (*Drug Price Information System*), RIPS (*Individual Health Service Provision Records*), and the SOAT (*Mandatory Traffic Accident Insurance*) tariff manual, were used to estimate prices. As recommended by IETS, drug costs were calculated using the price per minimum concentration unit for each medication.^21^ Additionally, local oncologists were consulted to validate the data sources and assumptions identified in the literature.

Specifically, the microcosting approach involved the identification, quantification, and valuation of individual healthcare resources associated with each health state. Drug acquisition costs included the study treatment, estimated per cycle based on nationally approved and recommended dosing regimens (eg, fixed dosing or weight-based dosing in mg/kg), and valued using nationally reported prices. Administration costs covered infusion-related procedures, valued per administration event using a standardized national procedure code from the RIPS database. Monitoring costs included routine laboratory tests (eg, complete blood count, liver function tests, serum creatinine, among others), imaging studies (eg, ultrasound, mammography, computed tomography, among others), and follow-up medical consultations (medical oncology, breast surgery, radiation oncology, among others), with frequencies defined according to clinical protocols and validated by local oncologists. Unit costs were derived from national tariff manuals and databases, using average reported prices where applicable, and applied consistently across all model cycles.

### Health State Utilities

Within the model, different sets of utility values can be applied. The first set is based on utility values derived from the KATHERINE trial EQ-5D data.^9^ The EQ-5D was administered at screening, during treatment, and every 6 months for 1 year after the study completion visit. Patient-reported outcomes (including the EQ-5D) were administered before any other study procedure was performed during the study visit. Similar utility values were seen across treatment arms; therefore, pooled utility values for both treatment arms were used in the model. Utility values are reported in **Table 1**.

### Adverse Events

Treatment-related adverse events were considered regardless of their timing or duration after drug administration. Based on consultation with local clinical oncologists, adverse events were reviewed according to their relevance for healthcare resource use in routine clinical practice. Clinical experts recommended focusing the modeling of adverse events on post-treatment, trial-related events classified under the “investigations” category in the KATHERINE trial updated results,^9^ as these represent explicit additional monitoring requirements in routine care. Accordingly, only the cost of an additional blood count in the T-DM1–treated population was included as an incremental cost. This approach was adopted to avoid overestimation of adverse event–related costs and is consistent with the reporting and categorization of adverse events in the KATHERINE trial.^9^

### Economic and Uncertainty Analysis

Robustness of model outcomes was evaluated through deterministic (one-way) and probabilistic sensitivity analyses. The probabilistic sensitivity analyses applied 1000 Monte Carlo simulations, using parameter distributions derived from clinical data or reasonable assumptions when required. Namely, utilities followed a gamma distribution; treatment effect was modeled based on a multivariate normal distribution; and adverse events, supportive care costs, and administration costs used a log-normal distribution. Each parameter was varied within its defined upper and lower bounds. The model generated mean costs, QALYs, and incremental cost-effectiveness ratios to capture decision uncertainty across varying parameters.

## RESULTS

### Base Case Analysis

The results of the cost-effectiveness analysis are shown in **Table 2**. In the base-case analysis, T-DM1 provided an additional 1.02 QALYs and generated cost savings of US $3192 per modeled patient. Given a WTP threshold of US $6831 for the Colombian health system, T-DM1 is considered a dominant and cost-effective alternative to trastuzumab. Additional details on life-years and QALYs gained across health states are summarized in **Supplementary Table S2**.

**Table 2.** Cost-Effectiveness Analysis of Trastuzumab-Emtansine vs Trastuzumab in Colombia

**Treatment**	**Cost (2024 US $)**	**Δ Cost vs Trastuzumab**	**QALYs**	**Δ QALYS vs Trastuzumab**	**ICER vs Trastuzumab**	**Net Monetary Benefit**
Base case						
T-DM1	110 096.4	–	10.95	–	–	–
Trastuzumab	113 288.1	-3191.7	9.93	1.02	Dominant	10 130.3
Probabilistic sensitivity analysis						
T-DM1	110 649.3	–	10.86	–	–	–
Trastuzumab	114 347.6	-3698.3	9.84	1.02	Dominant	10 651.7

Abbreviations: ICER, incremental cost-effectiveness ratio; QALY, quality-adjusted life-years; T-DM1, trastuzumab-emtansine.

1 US $ = 4071.35 COP.

As shown in **Table 3**, mean costs for T-DM1 were greater than trastuzumab in the adjuvant residual invasive disease state, reflecting acquisition costs. However, consistent savings were observed in all subsequent health states, leading to savings of up to US $30 510 per patient in metastatic treatment lines. Supportive care includes all health resources required after having a recurrence or metastasis, this includes diagnostic imaging, lab tests, medical consultations, other types of treatment (ie, radiotherapy or surgery) and any other drug used to treat recurrences or metastasis.

**Table 3.** Components of the Analysis of Trastuzumab-Emtansine vs Trastuzumab in Colombia (2024 US $)

**Treatment**	**T-DM1**	**Trastuzumab**	**Incremental Cost vs Trastuzumab**
Mean cost in residual invasive disease state			
Acquisition	44 197.22	9597.18	34 600.05
Diagnostic test	506.99	505.88	1.11
Drug administration	787.93	791.86	−3.93
Adverse events treatment	3.11	–	3.11
Supportive care	9518.69	8890.33	628.36
Total residual invasive disease state cost	55 013.95	19 785.25	35 228.70
Mean cost in non-metastatic recurrence state			
Supportive care	918.50	2335.10	−1416.60
Mean cost remission cost state			
Supportive care	49.51	126.03	−76.52
Mean cost 1L mBC			
Supportive care	45 508.29	76 018 39	−30 510.10
Mean cost 2L+ mBC			
Supportive care	8486.39	14 803.69	−6317.30

Abbreviations: 1L mBC, first-line metastatic breast cancer; 2L+ mBC, subsequent line metastatic breast cancer; T-DM1, trastuzumab-emtansine.

1 US $ = 4071.35 COP.

The clinical benefit of T-DM1 is the main driver of its economic value, resulting from the prevention of metastatic and locoregional events and deaths. This effect is evident early in the model, with 3-year reductions of 64% in locoregional events, 49% in metastatic events, and 46.9% in mortality. The prevention of locoregional and metastatic events remains consistently high and stable over a 30-year horizon. Details on the proportion of events avoided across the time horizon are presented in the **Supplementary Table S3**.

### Deterministic Analysis

**Figure 2.**
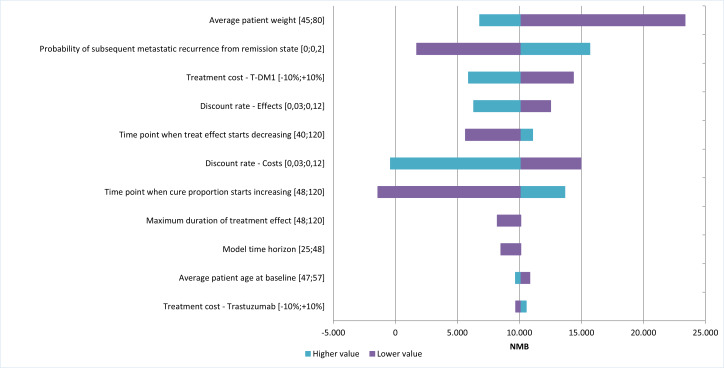
Tornado Diagram Abbreviations: NMB, net monetary benefit; T-DM1, trastuzumab-emtansine.

**Figure 2** shows the results of the univariate analysis, presented as the net monetary benefit (NMB) of T-DM1 vs trastuzumab, under the assumption that T-DM1 is a dominant strategy. The variables with the greatest impact on the NMB were patient weight, the probability of metastatic recurrence after remission, and the acquisition cost of T-DM1. In all cases, NMB remained positive, even when their values varied. Negative NMB values were observed only when applying the highest discount rate for costs or extending the time to cure. The results of the univariate analysis on the NMB are shown in **Figure 2**.

**Figure 3.**
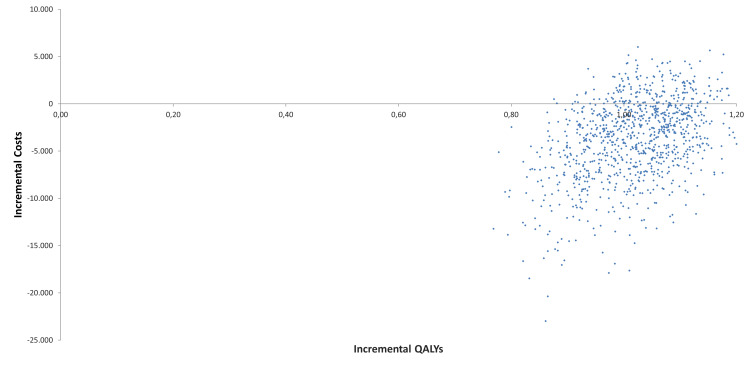
Cost-Effectiveness Plane Abbreviation; QALY, quality-adjusted life-year.

A total of 1000 Monte Carlo simulations were performed to evaluate the impact of parameter uncertainty using probabilistic distributions. The results of this analysis were consistent with those shown in the base case scenario. As shown in **Table 1**, T-DM1 remained a dominant alternative to trastuzumab in 81.2% of the simulations. The cost-effectiveness plane of the 1000 simulations is displayed in **Figure 3**, while **Supplementary Table S4** provides detailed results for life-years, QALYs, and costs with their respective distributions.

## DISCUSSION

Trastuzumab emtansine (T-DM1) is a recently approved option for patients with HER2+ eBC and residual disease following neoadjuvant treatment,^34^ demonstrating a significant reduction in recurrence and mortality compared with trastuzumab.^9^ This study assessed the cost-effectiveness of T-DM1 vs trastuzumab from the perspective of the Colombian National Health System, accounting for context-specific treatment alternatives, costs, and budget constraints.

Our findings show that T-DM1 is a dominant therapeutic strategy, delivering improved health outcomes while reducing overall costs over a 48-year horizon. This dominance is primarily explained by its ability to reduce recurrence rates and thereby limit the time patients spend in metastatic states. These reductions are especially relevant in the Colombian setting, where healthcare resource use and associated costs in metastatic disease are estimated to be 2 to 5 times higher than in early stages.^14^ These results underscore the importance of efficient resource allocation and highlight the value of economic evaluations in supporting the sustainability of national health systems.

When comparing our findings to those reported in cost-effectiveness evaluations performed in other settings, a similar trend was observed. In the United States, T-DM1 was also found to be dominant over trastuzumab.^25^ It showed that receiving T-DM1 resulted in 14.27 QALYs vs 12.49 with trastuzumab, which meant an incremental gain of 1.78 QALYs, along with cost savings of US $40 271. As in our analysis, these savings were primarily explained by a lower proportion of patients progressing to costly metastatic states (1L mBC, 2L+ mBC).

At a regional level, a similar analysis performed in Brazil showed that T-DM1 provided longer overall and progression-free survival, with gains of 2.7 and 6.0 years, respectively.^35^ In that study, T-DM1 was considered cost-effective under both the Brazilian and the US WTP thresholds over a 30-year time horizon. In our analysis, over a comparable time horizon, T-DM1 use was associated with a 12.8% reduction in deaths and a 60.0% reduction in locoregional events.

Despite its strengths, this analysis has several limitations. First, the model´s clinical inputs were mainly derived from the KATHERINE trial, which, while providing critical evidence on the impact of T-DM1 on disease progression and patients’ quality of life, may not fully capture the complexity and heterogeneity of real-world patient journeys. In the absence of effectiveness data from Colombia or Latin America, further research is needed to validate these findings using real-world data on treatment patterns, adherence, and recurrence rates in regional populations.

Another possible limitation relates to how disease recurrence pathways were represented in the model. Specifically, no explicit transition was modeled between non-metastatic recurrence and first-line metastatic BC. This approach reflects the structure of the KATHERINE trial,^36^ in which patients who developed metastatic disease within 2 months of a locoregional or contralateral recurrence were classified as having metastatic recurrence as their first disease event. As a result, the available evidence does not allow estimation of the risk of distant recurrence following a non-metastatic relapse. Given the absence of data to estimate metastatic risk following treatment for non-metastatic recurrence, explicitly modeling a mandatory transition between these states would require strong assumptions and could introduce greater bias than the approach adopted.

Importantly, patients experiencing a non-metastatic recurrence were still considered at risk of subsequent metastatic disease. Following a non-metastatic recurrence, patients were assumed to undergo a defined period of treatment and subsequently enter a remission state, during which they remained at risk of further disease progression and death. Any subsequent recurrence was assumed to be metastatic. This modeling approach is aligned with previously published economic evaluations in eBC, including the APHINITY cost-effectiveness model,^28,37^ and the risk of further progression was informed by external evidence.^32^ Nevertheless, uncertainty surrounding this assumption was explored through sensitivity analyses.

Another potential limitation of the analysis relates to the inclusion of adverse events in the model. Adverse events were selected based on their expected relevance for healthcare resource use in routine clinical practice and were validated with local clinical oncologists to reflect national management patterns for eBC. Excluding on-treatment adverse events and associated short-term disutility may underestimate the impact of treatment-related toxicity on patients’ quality of life. However, although on-treatment adverse events were more frequently reported in the KATHERINE trial,^36^ these events were considered clinically manageable and addressed within standard oncology care pathways, without generating sustained or long-term differential resource use between treatment arms in routine practice. In contrast, local oncologists identified post-treatment, trial-related adverse events (particularly laboratory abnormalities classified under the “investigations” category), as leading to explicit additional monitoring requirements. Consistent with the updated KATHERINE results,^9^ grade ≥3 and serious post-treatment, trial-related adverse events were reported in less than 1% of patients and occurred at similar rates across treatment arms, suggesting a limited impact on comparative costs and health outcomes. Accordingly, the model incorporated the cost of additional complete blood counts for patients receiving T-DM1, while other short-term adverse events and associated disutility were not explicitly modeled; nevertheless, their exclusion is expected to have a limited impact on the study findings.

Another limitation of this analysis is its reliance on a single clinical trial to inform key clinical inputs. Although the KATHERINE trial provides the most relevant evidence for patients with residual invasive HER2+ eBC treated in the adjuvant setting, dependence on a single trial may limit the generalizability of the findings. In this context, external validation of the model was constrained by the limited availability of long-term real-world evidence in this patient population. Nevertheless, the direction and drivers of the results are consistent with previously published cost-effectiveness analyses conducted in other settings, including the United States and Brazil,^25,35^ which similarly identified reduced progression to metastatic disease as the main contributor to long-term clinical and economic benefit. In addition, the model structure and key assumptions align with HTA-reviewed economic models in early HER2+ BC. Finally, preliminary observational data, such as the KARMA study,^38^ further provide supportive context for the clinical plausibility of the modeled outcomes, reporting favorable invasive disease-free outcomes and low recurrence rates with adjuvant T-DM1 in routine clinical practice. Although not designed to assess comparative effectiveness or long-term survival, these findings are consistent with the clinical patterns underlying the model and support the external plausibility of its key assumptions

Despite these limitations, the robustness of the findings was supported by multiple sensitivity analyses. In the deterministic sensitivity analysis, variations in key parameters, including changes in the acquisition costs of T-DM1 and trastuzumab, did not alter the conclusions. Even when trastuzumab was assumed to be less expensive or T-DM1 had higher acquisition costs, the results remained in favor of T-DM1. Similarly, scenarios including higher doses associated with increased patient weight did not modify the dominance of T-DM1. The probabilistic sensitivity analysis also confirmed that, despite uncertainty in model inputs and assumptions, T-DM1 remained dominant in over 80% of simulations, supporting the robustness of the results across a wide range of plausible scenarios.

Second, the analysis was conducted from a payer rather than a societal perspective, to minimize uncertainty associated with broader assumptions on productivity and social outcomes. However, incorporating elements such as productivity losses and caregiver burden could further strengthen the cost-effectiveness profile of T-DM1 in working-age populations.^39^

Overall, this study demonstrates that access to innovative therapies such as T-DM1 can have substantial clinical and economic benefits in middle-income countries like Colombia, particularly for patients with residual invasive HER2+ eBC. Although associated with higher initial costs, T-DM1 generates significant long-term cost savings by delaying, or preventing progression to advanced and costly disease stages, while improving patient outcomes. These findings support the use of T-DM1 in national treatment regimens under Colombia’s universal health coverage scheme, representing a strategic opportunity to optimize resource allocation, contain healthcare expenditures, and contribute to the growing body of evidence on the value of innovation in oncology.

## CONCLUSION

From the perspective of the Colombian Health System, T-DM1 is a cost-effective and dominant strategy for the adjuvant treatment of adult patients with residual invasive HER2+ eBC following neoadjuvant therapy. T-DM1 proved to be a dominant and cost-effective strategy overall, improving outcomes while reducing costs, mainly by lowering recurrence and progression. In Colombia, where metastatic disease is considerably more resource-intensive, these results emphasize the importance of adopting cost-effective innovations to ensure the sustainability of the health system.

### Disclosures

D.S.S., M.D.P., S.C.M., J.S., and L.P.P. work for Roche Colombia. D.B. has worked as a consultant for MSD and AstraZeneca and has received payment for lectures by MSD, BristolMyersSquibb, AstraZeneca, and Roche Colombia. M.L. has worked as a consultant, received payment for lectures, and participated in advisory boards for Roche Colombia.

## Supplementary Material

Online Supplementary Material
