# Supplementary material for: Cost-Effectiveness Analysis of Trastuzumab-Emtansine as Adjuvant Therapy for HER2-Positive Early Breast Cancer with Residual Invasive Disease in Colombia
Source: J Health Econ Outcomes Res. 2026 Feb 17;13(1):58–65. doi: 10.36469/001c.156056 (PMC12919760; doi:10.36469/001c.156056)
Supplement: Online Supplementary Material [file jheor_2026_13_1_156056_331405.pdf]

Journal of Health Economics and Outcomes Research

Online Supplementary Material

Cost-Effectiveness Analysis of Trastuzumab-Emtansine as Adjuvant Therapy for HER2-Positive Early Breast Cancer with Residual Invasive Disease in Colombia. *JHEOR*. 2026;13(1):58-65. doi:10.36469/jheor.2026.156056

Table S1: Summary of Invasive Disease-free Survival Parametric Distributions Information Criteria

Table S2: Summary of QALYs and Life-Years Gained by Health State

Table S3: Reduction in Events, %

Table S4: Probabilistic Sensitivity Analysis: Parameter Distribution and Results

This supplementary material has been provided by the authors to give readers additional information about their work.

This is an open-access article distributed under the terms of the Creative Commons Attribution 4.0 International License (CCBY-4.0). View this license's legal deed at http://creativecommons.org/licenses/by/4.0 and legal code at http://creativecommons.org/licenses/by/4.0/legalcode for more information.

Table S1. Summary of Invasive Disease-free Survival Parametric Distributions Information Criteria

Distribution	AIC	Rank	BIC	Rank
Exponential	1105.56	4	1110.17	2
Weibull	1107.55	5	1116.77	5
Log-normal	1098.36	1	1107.58	1
Gamma	1099.83	2	1113.67	4
Logistic	1104.06	3	1113.28	3
Gompertz	1107.56	6	1116.78	6

Abbreviations: AIC, Akaike information criteria; BIC, Bayesian information criteria.

Table S2. Summary of QALYs and Life-Years Gained by Health State

	Trastuzumab-Emtansine	Trastuzumab	Δ
QALYs (discounted)			
Residual invasive disease	10.476	9.048	1.428
Remission	0.086	0.220	-0.134
Non-metastatic recurrence	0.013	0.034	-0.020
Metastatic: Non-progressed	0.252	0.418	-0.166
Metastatic: Progressed	0.123	0.215	-0.092
Total	10.951	9.935	1.016
QALYs (undiscounted)			
Residual invasive disease	19.927	16.994	2.933
Remission	0.152	0.383	-0.230
Non-metastatic recurrence	0.016	0.041	-0.025
Metastatic: Non-progressed	0.355	0.586	-0.231
Metastatic: Progressed	0.188	0.323	-0.134
Total	20.640	18.327	2.313
Life-years (discounted)			
Residual invasive disease	13.443	11.608	1.834
Remission	0.111	0.282	-0.171
Non-metastatic recurrence	0.017	0.044	-0.026
Metastatic: Non-progressed	0.324	0.538	-0.214
Metastatic: Progressed	0.235	0.409	-0.175
Total	14.129	12.881	1.248
Life-years (undiscounted)			
Residual invasive disease	26.152	22.291	3.860
Remission	0.197	0.494	-0.297
Non-metastatic recurrence	0.021	0.053	-0.032
Metastatic: Non-progressed	0.399	0.626	-0.227
Metastatic: Progressed	0.279	0.437	-0.158
Total	27.186	24.209	2.977

Abbreviation: QALY, quality-adjusted life-year.

Table S3. Reduction in Events, %

Time Horizon	All Events (Excluding Deaths)	Deaths	Metastatic Events	Locoregional Events
3-year	50.7	46.9	49.0	64.0
4-year	49.1	46.1	46.8	63.1
5-year	48.1	44.7	45.5	62.6
10-year	46.2	37.1	43.4	60.8
15-year	46.2	31.2	43.5	60.2
20-year	46.3	25.4	43.7	60.0
30-year	46.5	12.8	44.0	60.0

Table S4. Probabilistic Sensitivity Analysis: Parameter Distribution and Results

Parameter	Distribution	T-DM1	Trastuzumab	Δ
Mean LYs	Log-normal			
In residual invasive disease on adjuvant treatment		13 443	11 608	1834
In non-metastatic recurrence		0.017	0.044	-0.026
In remission		0.111	0.282	-0.171
In metastatic		0.326	0.542	-0.216
In metastatic progression		0.237	0.414	-0.177
**Total LYs**		14 133	12.889	1244
**95% CI**		(14 054-14 237)	(12 727-13 107)	(1131-1327)
Mean QALYs	Gamma			
In residual invasive disease on adjuvant treatment		10 403	8984	1419
In non-metastatic recurrence		0.013	0.033	-0.020
In remission		0.084	0.214	-0.130
In metastatic		0.240	0.400	-0.159
In metastatic progression		0.123	0.214	-0.092
**Total LYs**		10 863	9845	1018
**95% CI**		(9826-11 188)	(8952-10 166)	(851-1161)
Mean total costs	Log-normal			
In residual invasive disease on adjuvant treatment		55 088	19 856	35 232
In non-metastatic recurrence		925	2351	-1426
In remission		50	127	-77
In metastatic		46 050	77 080	-31 031
In metastatic progression		8417	14 714	-6297
**Total LYs**		120	220	-100
**95% CI**		110 649	114 348	-3698

Abbreviations: CI, confidence interval; LY, life-year; QALY, quality-adjusted life-year; T-DM1, trastuzumab-emtansine.
